# Agreement and accuracy of radiographic assessment using a decision aid for medial Oxford partial knee replacement: multicentre study

**DOI:** 10.1186/s43019-022-00140-8

**Published:** 2022-03-14

**Authors:** Takafumi Hiranaka, Ryosuke Furuhashi, Kenichiro Takashiba, Takao Kodama, Kazuhiko Michishita, Hiroshi Inui, Eita Togashi

**Affiliations:** 1 Department of Orthopaedic Surgery and Joint Surgery Centre, Takatsuki General Hospital, 1-3-13 Kosobe, Takatski City, Osaka, 569-1192 Japan; 2 Department of Orthopaedic Surgery, Japanese Red Cross Hamamatsu Hospital, 1088-1 Kobayashi, Hamakita-ku, Hamamatsu City, Shizuoka 434-8533 Japan; 3 Department of Joint Reconstruction Center, Souseikai Fukuoka Mirai Hospital, 3-5-1, Kashiiteriha, Higashi-ku, Fukuoka City, Fukuoka 813-0017 Japan; 4 Department of Orthopaedic Surgery, Japan Community Healthcare Organization Saitama Medical Center, 4-9-3 Kitaurawa Urawa-ku, Saitama, 330-0074 Japan; 5 Department of Orthopaedic Surgery, Japan Community Healthcare Organization Yugawara Hospital, 2-21-6 Chuo, Yugawara, Kanagawa 259-0396 Japan; 6 Department of Orthopaedic Surgery, Faculty of Medicine, The University of Tokyo, 7-3-1, Hongo, Bunkyo-ku, Tokyo, 113-0033 Japan; 7 Department of Orthopedic Surgery, Yamagata Tokushukai Hospital, 2-3-51 Kiyozumimachi, Yamagata city, Yamagata 990-0834 Japan; 8 Department of Orthopaedic Surgery and Joint Surgery Centre, Takatsuki General Hospital, 1-3-13, Kosobe-Cho, Takatsuki City, Osaka, 561-1115 Japan

**Keywords:** Operation, Indication, Unicompartmental knee arthroplasty, Radiography

## Abstract

**Background:**

Indication for mobile-bearing partial knee replacement (PKR) is made on the basis of a radiological decision aid. This study aimed to reveal the inter-rater reproducibility and accuracy of the decision aid when used by experienced surgeons.

**Patients and methods:**

Anonymised radiographic image sets (anteroposterior, lateral, varus/valgus stress in 20° knee flexion, and skyline views) from 20 consecutive patients who underwent knee replacement were assessed by 12 experienced surgeons. Agreements of each section and accuracy were compared by intra-operative inspection of the status of the anterior cruciate ligament (ACL) and medial and lateral cartilage according to the protocol of Radiographic Assessment for Medial Oxford PKR. Fleiss’ kappa (*κ*) values were used as a statistical measure.

**Results:**

Full-thickness medial cartilage had the best agreement between the surgeons (*κ* = 94.7%) and best accuracy (94.2%). Although functioning ACL (90.8%), intact cartilage (91.7%) and full-thickness lateral cartilage defects (86.1%) were accurately diagnosed, diagnoses of deficient ACL (up to 42.5%) and partial-thickness lateral cartilage defects (11.7%) were poor; they were sometimes misdiagnosed as being intact. Moreover, agreement of lateral and valgus stress radiographs regarding intact MCL function, as well as the overall decision, was considered to be inadequate (*κ* = 0.47, 0.58 and 0.51, respectively).

**Conclusions:**

Although the radiological aid is useful for selection of patients who are likely to be suitable for PKR, surgeons should still carefully assess the lateral weight-bearing area for partial-thickness loss and deficiency of the ACL because they were sometimes overlooked by surgeons using radiographs. MRI will be helpful to improve the accuracy of determination of Oxford PKR indication.

## Introduction

There is increasing interest in partial knee replacement (PKR) as it has been reported to be an efficient treatment option for knee osteoarthritis (OA). Advantages over total knee replacement (TKR) include faster recovery, deeper flexion angle, fewer systemic complications and reduced mortality, as well as superior patient satisfaction [1–3]. On the other hand, national registry data revealed that the revision rate was higher for PKR than for TKR, even after adjustment for the pre-operative conditions [2]. Patient selection is reported to be the key to successful PKR. Intact lateral cartilage and intact anterior cruciate ligament (ACL) are necessary conditions for successful application of PKR [4]. In addition to this, Kozinn and Scott [5] proposed a strict indication recommending that patients over 82 kg, younger than 60 years, who are extremely physically active or who perform heavy labour, or who have chondrocalcinosis or exposed bone in the patellofemoral joint should be contraindicated. As a result, the usage of PKR has been reported to be just 9.3% in Sweden [6], 8% in the USA [7], 11.2% in the United Kingdom [8] and 5.4% in Australia [9]. However, these indications are for fixed-bearing PKR. Elsewhere, Liddle et al. reported that the best result could be achieved when the usage of PKR ranged between 40% and 60% of all cases of knee arthroplasty [10]. As PKR has been assumed to be a technically demanding operation, increasing the number of PKRs under validated indication could improve the post-operative survivorship after PKR [11], this difference can be achieved by patient selection. In their examination of the best indication for PKR, White et al. introduced the concept of anteromedial osteoarthritis (AMOA) to show indication for mobile-bearing PKR [12]. Knees that have bone-on-bone (full-thickness cartilage defect) in the medial compartment, intact cartilage in the lateral compartment, a functionally normal medial collateral ligament (MCL) and anterior cruciate ligament (ACL), and acceptable patellofemoral joint arthritis are considered to have AMOA [4, 13]. AMOA can reportedly be detected by plain varus and valgus radiographs. A radiological decision aid was introduced to enable sophisticated decision-making regarding mobile-bearing PKR [14]. Hamilton et al. reported that clinical results were slightly better in patients who met the criteria compared with those who did not, and that approximately half of all patients met the criteria [15]. The decision was made by a single senior surgeon, however, and its interrater reliability was not evaluated in the paper. In addition, the accuracy of the decision aid was not described. This study aims to reveal the inter-rater reproducibility and accuracy of the decision aid when used by experienced surgeons.

## Materials and methods

### The radiological decision aid

The decision aid contains five criteria based on plain anteroposterior and lateral radiographs along with varus and valgus stress radiographs at 20° flexion [14]:

1. *Space between the femoral and tibial bony surface*
  Medial bone-on-bone is evaluated on varus radiographs and indicates a full-thickness cartilage defect in the medial compartment. A space between the femoral and tibial bony surface can imply a partial-thickness cartilage defect that would rule out PKR.
2. *Functionally intact ACL*
  The location (if present) of a bony erosion may be seen on lateral radiographs. When the ACL is intact, the erosion is located anteriorly or is not seen. If it locates and/or extends posteriorly, the ACL would be deficient [16].
3. *Full-thickness lateral cartilage*
  Lateral cartilage thickness was assessed on valgus stress radiographs. We considered the lateral cartilage to be intact if the lateral joint space was fully retained. Any osteophytes on the lateral condyle were ignored because their existence has not been reported to affect the clinical outcome [17].
4. *Functionally normal MCL*
  If the MCL is functional and not contracted, it retains its original length and any varus deformity will be correctable; this also implies that the ACL is intact [12]. If the ACL is intact, although the MCL will shrink during knee extension because of cartilage wear, its length can be restored in knee flexion, when the condyles with intact cartilage thickness are facing each other. Consequently, the MCL never contracts and the varus is therefore correctable. On the contrary, in the case of an ACL deficiency, the tibia moves forward and the cartilage of the posterior tibial plateau could be worn out. The exposed bony surfaces contact each other even in the knee flexion position, and eventually the MCL will be shortened; the varus is therefore not correctable. Such knees are unsuitable for PKR.
5. *Acceptable patellofemoral joint*
  Medial facet OA, with or without bone loss, and lateral facet OA without bone loss are accepted. Lateral facet OA with bone loss, grooving or subluxation is unacceptable [18].
6. *Overall decision on PKR suitability*
  All sections are rated “yes” or “no”. Knees with “yes” for all sections were considered to be suitable for mobile-bearing PKR; they were otherwise rated as unsuitable.

### Multicentre study

This study was approved by the institutional review board of our hospital, and written informed consent was obtained from all patients. This study included 20 consecutive patients who underwent single-sided total knee replacement (TKR) or (PKR) in the corresponding author’s hospital in August 2019. Pre-operative radiography sets including anteroposterior (AP), true lateral, valgus and varus stress at 20° flexion and skyline view were anonymised and prepared.

We recruited 12 experienced surgeons with extensive knowledge and skills who are domestic instructors in the use of mobile-bearing PKR. The average years of experience of surgery and average number of PKRs performed per year of the participating surgeons were 26.3 years (15–43 years) and 72.5 cases (24–150 cases), respectively. The radiographic datasets were sent electronically to the participants, and rating was performed in each hospital. The reviewers rated “yes” or “no” for each section on the basis of the 20 radiograph sets, and an overall decision of suitability or non-suitability for PKR was made on the basis of the rates of the five sections. The results were sent back to the corresponding author’s hospital for analysis.

The condition of the ACL and the cartilage at the medial femoral condyle, medial tibial plateau and lateral femoral condyle (weight-bearing area) was assessed intra-operatively (Table 1). The assessment was done by the corresponding author and another assistant surgeon. If their diagnoses differed, re-assessment was performed. If the decision was still different, the corresponding author’s decision was used for analysis. If the ACL was graded as normal, with synovial damage or with longitudinal split, it was considered to be functioning; it was otherwise classified as deficient [19]. If the cartilage was rated as normal or as having superficial damage, it was deemed to be intact; it was otherwise rated as defective. Regarding the lateral femoral condyle, a full-thickness cartilage defect at the lateral edge was ignored because the lesion was in the non-weight-bearing area. Radiographic decisions were validated on the basis of intra-operative findings.

**Table 1:** Status of ACL and lateral cartilage

ACL status		Lateral cartilage status	
Normal	ACL functioning (ACLF)	Normal	Intact
Synovial damage		Superficial damage	
Longitudinal split		Partial-thickness defect	Defective
Friable and fragmented	ACL deficient (ACLD)	Full-thickness defect	
Absent		Bone loss	

The accuracy of the estimation of the status of medial femoral and tibial condyles, lateral femoral condyles and ACL (functioning or deficient) was evaluated on the basis of intra-operative inspection, and the percentage of knees whose condition was correctly predicted was calculated. Regarding the lateral femoral condyle, the ability to diagnose it as intact or defective (partial, full-thickness or both) was assessed.

### Statistical analysis

Fleiss’ kappa values were calculated to evaluate the reliability of agreement between the raters. Calculations were performed using Microsoft Excel (Microsoft Corp., Redmond, WA). The predictability of ACL status, lateral cartilage status and overall PKR suitability were compared using repeated measures analysis of variance followed by Bonferroni’s multiple comparison test. Moreover, Pearson’s correlation coefficient between the predictabilities and surgeons’ years of experience and the number of surgeries were calculated. Analysis was performed using easy R (EZR; Jichi Medical University, Japan) running on R (R Foundation for Statistical Computing, Vienna, Austria) [20].

## Results

On the basis of intra-operative inspection, 8 of the 20 cases were considered suitable for PKR (40%). Interestingly, the percentage of knees which the respective raters considered to be suitable for PKR varied between 45% and 75% with a mean of 60%, showing a higher percentage than the suitability based on macroscopic findings. These values were not significantly correlated with the rates of PKR usage (*r* = 0.09, *P* = 0.77).

**Fig. 1:**
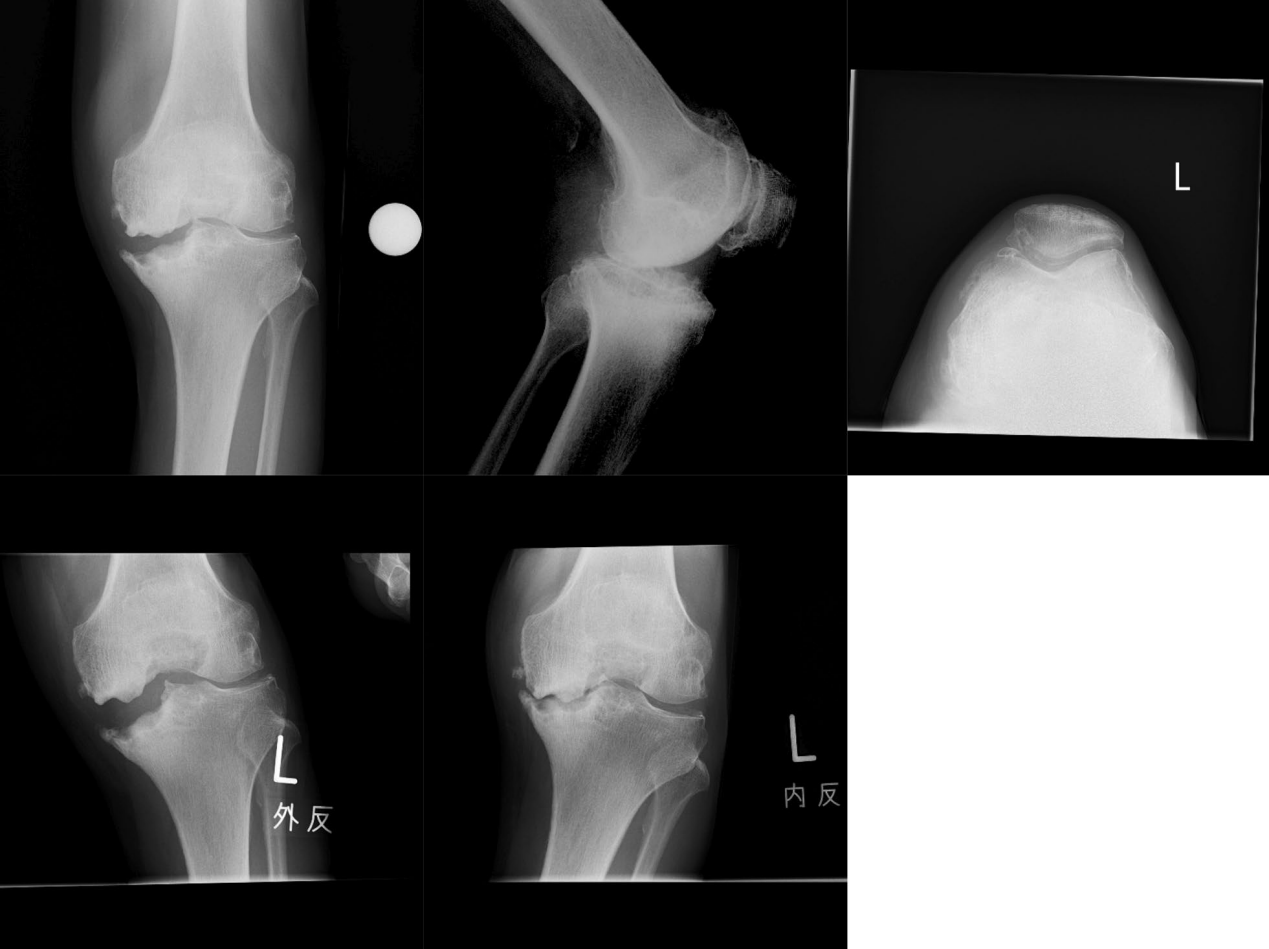
An extreme case (case 20). Despite the severe bone loss in the medial compartment, the ACL and the lateral cartilage were intact. Some doctors judged this case as having no medial bone-on-bone appearance

The details of the decisions based on the radiographs and the intra-operative findings are presented in Table 2. The accuracy of the diagnosis of the respective sections is summarised in Table 3. The most accurately diagnosed section was medial bone-on-bone, indicating full-thickness cartilage loss on both condyles. Although a full-thickness defect of the medial condyle was detected in every case intra-operatively, two cases were not outlined as bone-on-bone. These cases had a severe loss of bone with grooving in the medial tibial plateau. Consequently, there was still some space between the bones due to non-conformity between the articular surfaces (Fig. 1).

**Table 2:** Details of the decision for each section in each case

Case	BnB	ACL	LC	MCL	PFJ	Overall	ACL status	Lateral cartilage	PKR suitability	Correct diagnosis (%)
1	12	12	7	11	10	8	Functioning	Normal	Yes	67
2	12	2	2	9	4	0	Deficient	Full-thickness defect	No	100
3	12	12	12	12	12	12	Deficient	Normal	No	100
4	12	7	12	10	12	7	Functioning	Normal	Yes	58
5	12	12	7	9	12	7	Deficient	Partial-thickness defect	No	42
6	12	12	12	12	10	11	Deficient	Partial-thickness defect	No	8
7	12	7	11	12	10	7	Deficient	Partial-thickness defect	No	42
8	12	8	11	12	12	9	Functioning	Superficial damage	Yes	75
9	5	6	3	7	10	0	Deficient	Full-thickness defect	No	100
10	12	4	10	10	12	3	Deficient	Superficial damage	No	75
11	12	11	12	12	12	11	Functioning	Superficial damage	Yes	92
12	12	11	12	12	12	11	Functioning	Normal	Yes	92
13	12	12	12	12	12	12	Functioning	Normal	Yes	100
14	12	12	12	12	12	12	Functioning	Normal	Yes	100
15	12	11	12	12	12	12	Functioning	Normal	Yes	100
16	12	7	11	9	11	6	Deficient	Superficial damage	No	50
17	12	9	11	8	6	5	Functioning	Partial-thickness defect	No	58
18	12	12	12	11	12	11	Deficient	Partial-thickness defect	No	8
19	12	5	0	8	5	2	Deficient	Full-thickness defect	No	83
20	5	4	9	8	11	0	Functioning	Normal	Yes	0

BnB, medial bone on bone; ACL, functionally intact ACL; LC, intact lateral cartilage; MCL, functionally intact MCL; PFJ, acceptable patellofemoral joint change

**Table 3:** Accuracy of diagnosis of the intra-operative inspection

Joint status	Radiographs used	Accuracy (%)
Medial bone-on-bone	Varus stress	94.2
ACL functioning	Lateral	80.8
	Valgus stress	90.8
	Lateral + valgus stress	72.5
ACL deficient	Lateral	34.2
	Valgus stress	17.5
	Lateral + valgus stress	42.5
Normal lateral cartilage	Valgus stress	91.7
Lateral cartilage defect	Valgus stress	39.6
Partial-thickness defect	Valgus stress	86.1
Full-thickness defect	Valgus stress	11.7

A functioning ACL showed good rates of diagnosis based on valgus stress radiographs (90%) followed by lateral radiographs (80%). In contrast, ACL deficiency was not sufficiently diagnosed using lateral radiographs (34%) or valgus stress radiographs (18%). If the combination of both radiographs was used to make the decision, the accuracy increased to 43%, but this was still inadequate.

Regarding the lateral cartilage, intact lateral cartilage was well diagnosed (92%), while lateral cartilage defects were not accurately detected (40%). Although full-thickness defects were well outlined (86%), partial-thickness defects were poorly recognised (12%).

The overall agreement of the suitability of a case for PKR was moderate (*κ* = 0.51). The best agreement was found in the medial bone-on-bone section, followed by full-thickness lateral cartilage on valgus stress radiographs, and acceptable change in patellofemoral disease on skyline view. The functioning ACL showed inadequate agreement (*κ* = 0.47) (Table 4).

**Table 4:** Inter-rater agreements of decisions for each section

Criterion	Kappa	95% CI	Overall agreement (%)
Medial bone-on-bone	0.89	0.75–1.00	94.7
Functionally intact ACL	0.47	0.26–0.67	77.3
Full-thickness lateral cartilage	0.68	0.52–0.85	84.2
Functionally normal MCL	0.58	0.40–0.77	79.2
Acceptable patello-femoral joint	0.69	0.51–0.86	84.3
Overall PKR indication	0.51	0.31–0.70	75.3

**Fig. 2:**
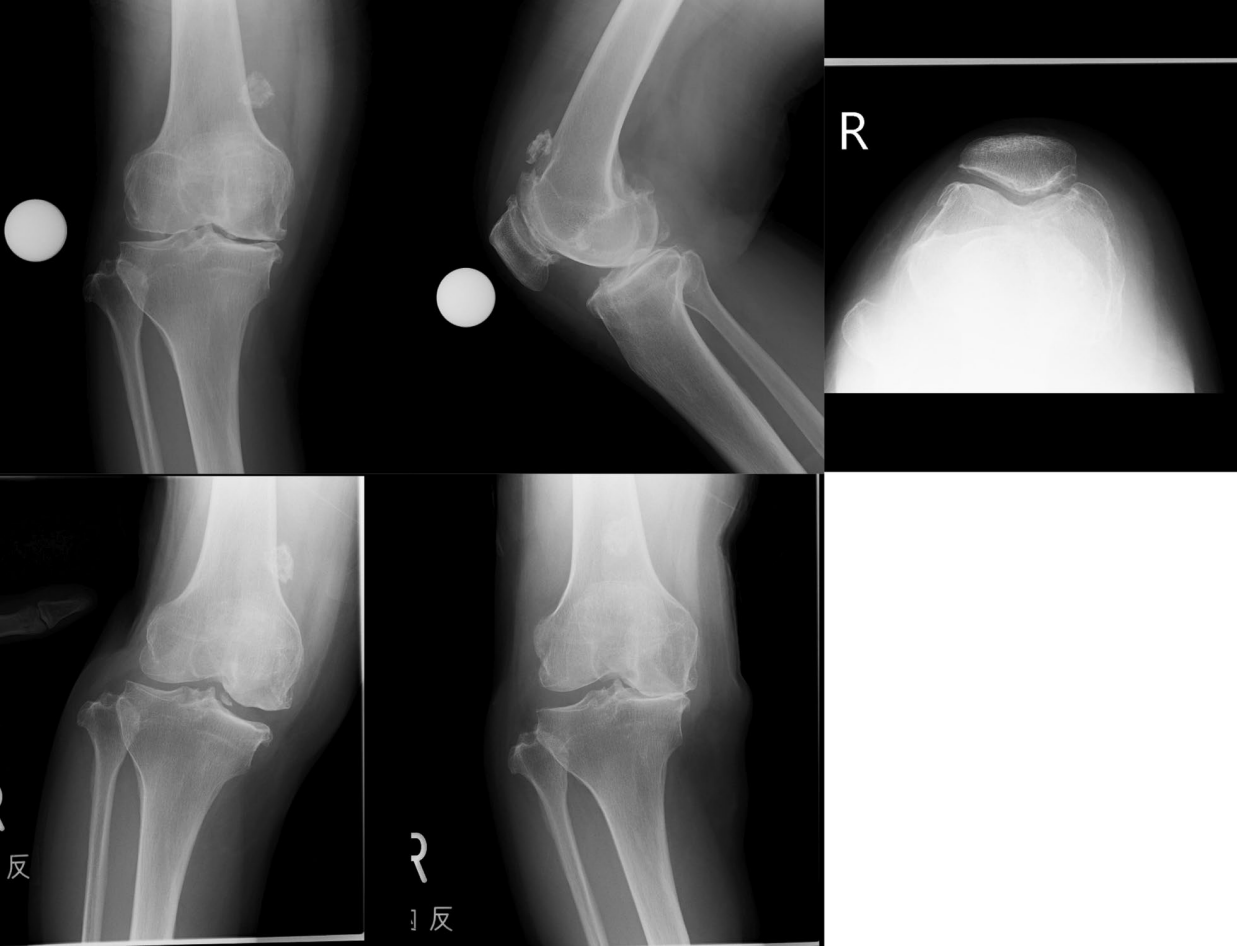
A misleading case (case 6). Most surgeons diagnosed no erosion and correctable varus along with retained lateral cartilage, despite a deficient ACL and the presence of a partial-thickness defect of the lateral femoral condyle

**Fig. 3:**
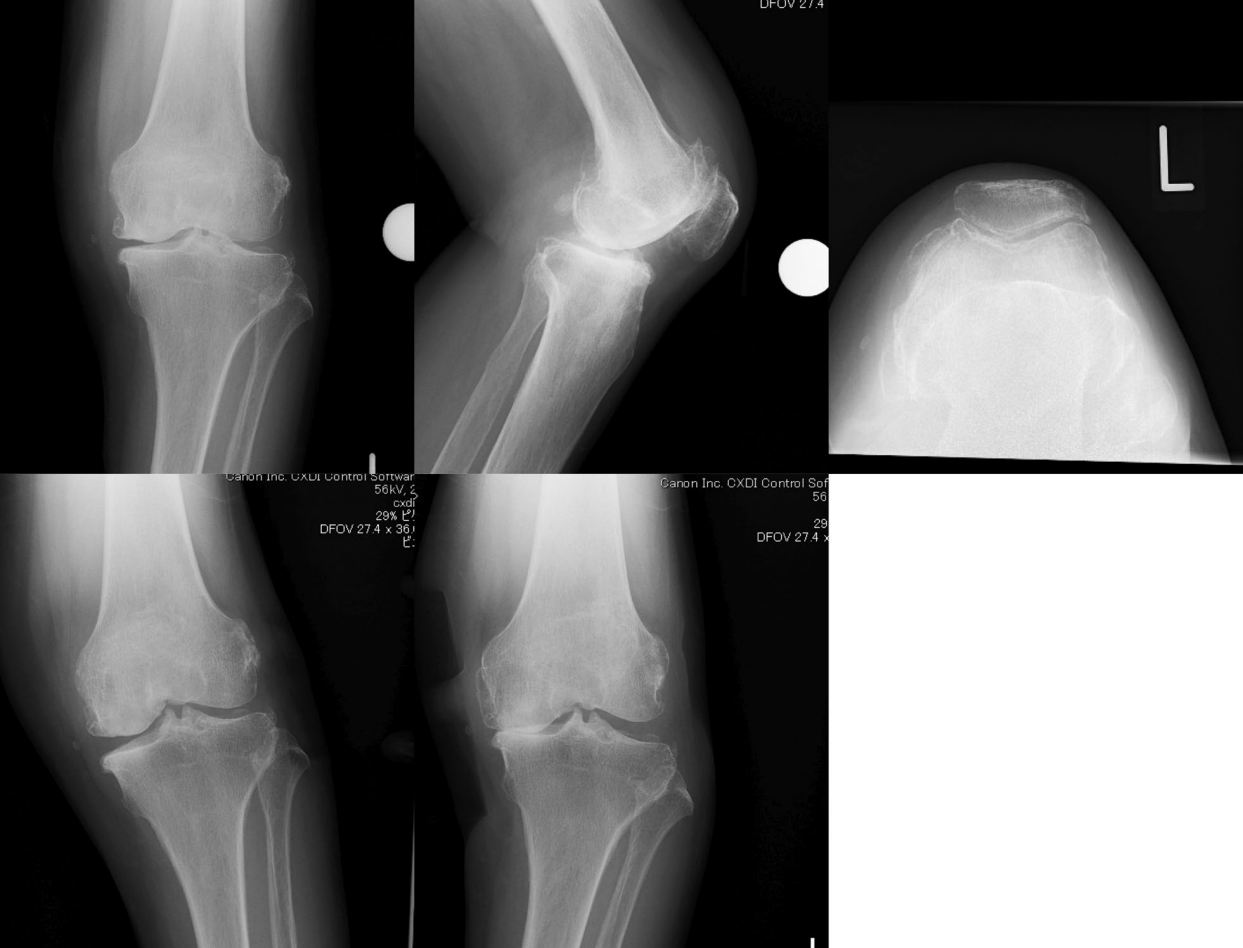
Another deceptive case (case 18). The erosion seemed to locate anteriorly. The lateral cartilage appeared normal and varus looked well corrected. The ACL was also deficient, and lateral cartilage was partially defective

Details of each subject are presented in Table 3. On the whole, suitable cases were well diagnosed except for one extreme case, which was unsuitable for PKR (Fig. 1), despite a functioning ACL and lateral cartilage. On the other hand, unsuitable cases had a tendency to be diagnosed as suitable, mainly due to thickness cartilage defects of the lateral condyle being only partial, or a deficient ACL (Figs. 2, 3).

The percentage of correctly predicted suitability varied between surgeons (45% to 75%). There were no significant correlations of the predictability with experience as a surgeon, annual number of TKA and PKR performed, and percentage of PKR usage (0.130, −0.098 and −0.025, respectively). The predictably of ACL status and lateral cartilage status varied between 50–70% and 65–75%, respectively. Similarly, the predictability of ACL and lateral cartilage status did not show significant correlation with experience or amount of surgery. The lateral cartilage status was significantly better predicted than ACL status and overall status (versus ACL status *P* < 0.001, versus overall PKR suitability *P* = 0.01).

## Discussion

This is the first report concerning agreement of the indication of PKR between surgeons. Some reports suggest that only 5–8% of candidates for knee arthroplasty are eligible for PKR [21, 22]. On the other hand, Hamilton et al. [15] reported that around 50% of patients were found to be suitable for PKR using a decision aid. However, the decision was made by a single rater in their study, meaning there was insufficient evaluation of inter-rater variation. In our study, 12 experienced surgeons were recruited to perform the evaluation. Despite the expertise of the raters, the overall PKR decision rate varied between 45% and 75%. Interestingly, the rate was higher than the actual rate of PKR suitability in this case series (40%). This variation might be caused by the accuracy of diagnosis and inter-rater variations.

Regarding the accuracy, all raters scored a higher percentage of the knees suitable for PKR compared with the actual intra-articular condition (the status of the ACL and lateral cartilage). Although functioning ACLs were accurately detected with excellent agreement, deficient ACLs were sometimes misdiagnosed as functioning on both lateral and valgus radiographs. This indicates that ACL deficiency would be correctly diagnosed. However, even if a radiograph shows an ACL as functioning, it is possible that the surgeon may find ACL deficiency during the operation. Mancuso et al. reported that there are two types of pathology regarding OA and ACL deficiency: primary ACL deficiency followed by secondary OA, and secondary ACL rupture caused by developed primary OA [23]. The latter can maintain the characteristics of OA with a functioning ACL despite the deterioration of the ACL. Such a deficiency is difficult to recognise using functional radiographs.

Although full-thickness defects of the lateral cartilage were accurately diagnosed, partial-thickness defects of the lateral cartilage have been poorly detected on pre-operative radiographs [24]. Waldstein et al. reported that valgus stress radiographs did not predict cartilage degeneration, and the Osteoarthritis Research Society International (OARSI) macroscopic grade did not correlate with the lateral cartilage thickness apparent on valgus stress radiographs [25]. It is understandable that contacting the surrounding full-thickness cartilage area hides partial-thickness defects under valgus stress conditions. In addition, a lesion might be located in a minimal area, and consequently, the defect can be detected only when each (femur and tibial) lesion meets during the stress radiograph. Consequently, the presence of a normal lateral joint space does not always confirm intact lateral cartilage. MRI could be helpful to evaluate the lateral cartilage as well as the condition of the meniscus. Although it can exaggerate the disease and incurs additional cost, it can improve the accuracy of the patient selection for PKR and could diminish the failure rate [17].

We found insufficient inter-rater agreement regarding the lateral radiographs, which is a reflection of the ACL condition. Keyes et al. reported that, if there is no posterior tibial erosion or the erosion is not seen on the lateral radiographs, there is a 95% probability that the ACL would be functionally intact [16]. However, as the decision aid does not mention how to judge a case without obvious erosion, this can cause some misinterpretation. In addition, it is not always easy to recognise erosion owing to image quality or overlapping condyles. The boundary of the erosion, and whether or not it is located in the posterior region, is not clear. More quantitative criteria based on large numbers of clinical cases will be needed. Again, MRI could improve the accuracy of the ACL status evaluation, if it is available and the cost is tolerable for patients. Further study might be valuable in which the accuracy and reliability of the patient selection for PKR is evaluated on the basis of not only radiographies, but also MRI.

Interestingly, the predictability of ACL and lateral cartilage status and overall PKR suitability was varied, between 45% and 75%. The lateral cartilage was more correctly predicted than overall suitability and ACL status, but decisions were not perfect. Moreover, the predictability was not influenced by the years of experience or volume of surgery. Although all raters were experienced surgeons, the decision is considered not to be affected by the surgeon’s familiarity with the surgery.

There are some limitations to this study. Firstly, the results may change according to the selection of patients. Each patient has individual characteristics. This case series included an extreme case (Fig. 1) with a functioning ACL and lateral cartilage with severe bone loss and grooving. In addition, the percentage of the series suitable for PKR was lower than typical; in general, 50–60% of patients who are candidates for knee arthroplasty undergo PKR in our institution. However, we recruited patients prospectively to exclude selection bias. Secondly, there was no evaluation of inter-rater reproducibility. It is possible that a previously assessed case may affect the judgement of the next case, and another study is needed to assess this. Thirdly, the number of patients was relatively small (20 patients), and no power analysis was performed because the statistics were descriptive and no comparison was conducted in this study. Nevertheless, the limitation of decision-making using radiography was recognised by this study. Finally, the decision was made on the basis of only five types of radiographs. Some other radiographs, such as Rosenberg’s view or long-standing radiographs, are frequently used. A surgeon makes a decision comprehensively using additional information from both radiological and clinical investigations. Regarding the surgeons who participated in this study, 6 out of 12 perform MRI routinely and another 5 obtain it only in selected patients. Thus, in total, 11 out of 12 surgeons used MRI for decision-making at least in dubious cases. Most importantly, surgeons do not always undertake PKR, even if a patient is determined to be suitable for it. The reason for the individual surgeon’s decision in each case was not evaluated. Despite these limitations, this study provides valuable information for sophisticated decision-making in selection of suitable patients and improvement of PKR outcomes.

## Data Availability

No data or materials are provided.
